# Aberration-free volumetric high-speed imaging of *in vivo* retina

**DOI:** 10.1038/srep35209

**Published:** 2016-10-20

**Authors:** Dierck Hillmann, Hendrik Spahr, Carola Hain, Helge Sudkamp, Gesa Franke, Clara Pfäffle, Christian Winter, Gereon Hüttmann

**Affiliations:** 1 Thorlabs GmbH, Maria-Goeppert-Straße 9, 23562 Lübeck, Germany; 2 Institute of Biomedical Optics, University of Lübeck, Peter-Monnik-Weg 4, 23562 Lübeck, Germany; 3 Medical Laser Center Lübeck GmbH, Peter-Monnik-Weg 4, 23562 Lübeck, Germany; 4 Airway Research Center North (ARCN), Member of the German Center for Lung Research (DZL), Germany

## Abstract

Certain topics in research and advancements in medical diagnostics may benefit from improved temporal and spatial resolution during non-invasive optical imaging of living tissue. However, so far no imaging technique can generate entirely diffraction-limited tomographic volumes with a single data acquisition, if the target moves or changes rapidly, such as the human retina. Additionally, the presence of aberrations may represent further difficulties. We show that a simple interferometric setup–based on parallelized optical coherence tomography–acquires volumetric data with 10 billion voxels per second, exceeding previous imaging speeds by an order of magnitude. This allows us to computationally obtain and correct defocus and aberrations resulting in entirely diffraction-limited volumes. As demonstration, we imaged living human retina with clearly visible nerve fiber layer, small capillary networks, and photoreceptor cells. Furthermore, the technique can also obtain phase-sensitive volumes of other scattering structures at unprecedented acquisition speeds.

Fourier-domain optical coherence tomography (FD-OCT) images living tissue with high resolution^1^,^2^,^3^. Its most important applications are currently in ophthalmology, where it compiles three-dimensional data of the human retina that are not attainable with any other imaging method. However, especially at high numerical aperture (NA), aberrations significantly reduce the resolution and the focal range restricts the volume depth of a single measurement. Currently, when imaging the retina, aberrations are either accepted at low NA or removed by using adaptive optics (e.g., refs 4 and 5). The disadvantages of limited focal range in OCT are reduced by using non-diffracting (Bessel) beams (e.g., ref. 6).

As an alternative without additional optical components, computational methods have been shown to remove aberrations and defocus^7^,^8^,^9^,^10^,^11^,^12^. However, these techniques require the phase of the backscattered light to be known. Therefore limitations occur when applying this to moving targets such as the human retina *in vivo*. One has to overcome two major challenges to make the technique broadly applicable to high resolution retinal imaging: First, volumes have to be acquired coherently, i.e., phases must not be influenced by sample motion, but must only depend on tissue morphology. Secondly, even higher order aberrations need to be determined reliably from the recorded data, requiring sophisticated numerical algorithms.

Essentially, FD-OCT acquires coherent volumes. It interferometrically detects backscattered infrared light at multiple wavelengths and computes its depth-resolved amplitudes and phases at one lateral position (A-scan). To obtain a three-dimensional volume one usually acquires A-scans for different lateral positions by confocal scanning. If all A-scans are measured without sample motion, the volume is phase-stable and coherent, and the advantage of such data was previously shown: degradation of the lateral resolution by a limited focal depth was eliminated by interferometric synthetic aperture microscopy (ISAM)^9^. Later, Adie *et al.*^10^,^11^ corrected aberrations. Imaging an immobilized human finger tip finally demonstrated the assets of these techniques *in vivo* using scanned volumetric OCT^12^.

However, a rapidly moving sample, such as the human eye, destroys the phase stability and renders the acquired OCT dataset virtually not coherent, making ophthalmic applications of ISAM rather challenging^13^,^14^. Only by using sample tracking and extensive motion correction the achieved phase-stability allowed computational refocusing when imaging an unsupported finger^15^, and eventually Shermanski *et al.* succeeded in imaging the photoreceptor layer of living human retina^16^. Due to fast motion of the eye, sufficient phase-stability was achieved only when restricting imaging to a single *en face* layer of the retina, and when selecting frames with little tissue motion. Thus, even fast scanning OCT devices could not provide the necessary phase stability for computational correction techniques in full three-dimensional retinal imaging.

To acquire a sufficiently phase-stable three-dimensional volume of moving targets and to numerically correct defocus and aberrations, a further increase in imaging speed is required. In principle, full-field swept-source OCT (FF-SS-OCT)^17^ can acquire data several orders of magnitude faster than confocal OCT, as it removes the need for lateral scanning by imaging all positions onto an area camera. Furthermore, it allows higher radiant flux on the sample without damaging the tissue. So far, FF-SS-OCT showed poor image quality and the available cameras limit imaging speed and field of view^18^.

Here, we show that a remarkably simple FF-SS-OCT system obtains truly coherent three-dimensional tomograms of the living human retina with high image quality. Its acquisition speed is 38.6 million lateral points (A-scans) per second, which exceeds current clinical systems by several orders of magnitude and is about one order of magnitude faster than any other OCT system in a research environment^19^,^20^. This fast and phase-stable acquisition allows a computational optimization of image quality, similar to techniques in synthetic aperture radar^21^ and to a recently published method to correct aberrations in interferometric imaging^22^, which removes defocus and aberrations and backtraces all light to its three-dimensional scattering location in a volume spanning multiple Rayleigh lengths in depth. No additional hardware is needed to track motion or to determine and correct aberrations. To show the validity of this approach, we imaged the living human retina at maximum pupil diameter (7 mm), and obtained images of the nerve fiber layer, small vascular structures, and photoreceptor cells with nearly diffraction-limited resolution. The presented technique visualizes living and moving tissue with higher lateral and temporal resolution than previously possible. In addition, the detected phase of the scattered light can provide valuable data on small sub-wavelength changes in axial direction. Thereby the technique can visualize dynamic *in vivo* processes that were previously inaccessible.

## Data Acquisition and Processing

**Figure 1:**
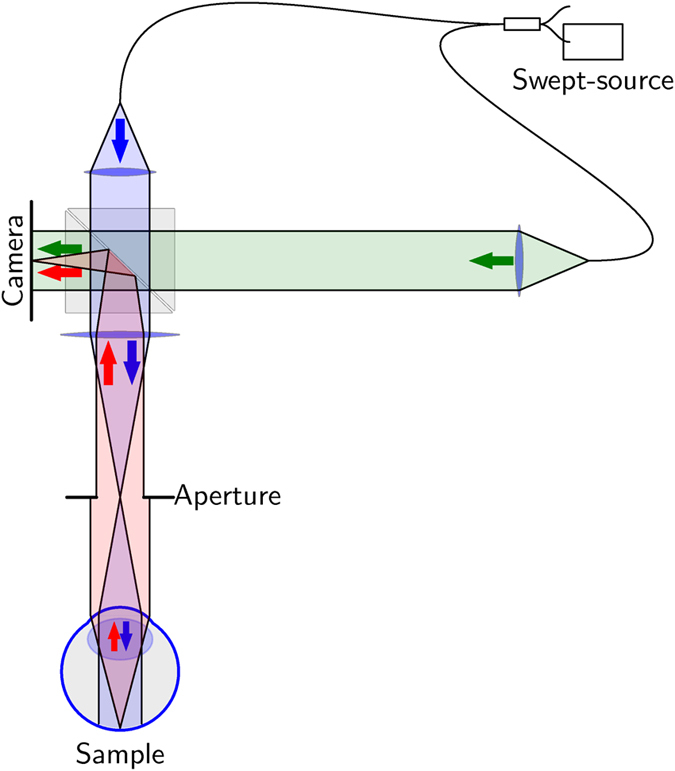
Setup of the full-field swept-source OCT for retinal imaging. Light from a tunable light source is split into reference (green) and sample arm (blue); the sample light illuminates the retina and the backscattered light (red) is imaged onto the camera where it is superimposed with the reference light.

To acquire an entire three-dimensional volume coherently by FF-SS-OCT, interference images of light backscattered from the retina and reference light were acquired at multiple wavelengths. To this end, the interference pattern was generated in a simple Mach-Zehnder type setup (see Methods) as shown in Fig. 1. For a single volume, a high-speed camera (Photron FASTCAM SA-Z) recorded 512 of these interferograms during the wavelength sweep of a tunable laser (Superlum Broadsweeper BS-840-1), covering 50 nm and centered at 841 nm. Images were acquired with 896 × 368 pixels at 60,000 frames per second, which results in an acquisition rate of 117 volumes per second and corresponds to 38.6 MHz A-scan rate and 9.9 GHz voxel rate. During each measurement, 50 successive volumes were acquired to allow averaging and thus to increase image quality after reconstruction. For a single volume, the sensitivity of this full-field setup was evaluated to about 75 dB.

We first reconstructed the acquired data using standard OCT processing (see Methods). Most importantly, a Fourier transform of the acquired data along the wavenumber axis reconstructed the sample volume. Afterwards computational corrections of axial motion maximized image quality; this was inevitable, even at these acquisition rates. Each resulting image volume was coherent and contained the correct phases, but still suffered from reduced quality due to a limited focal depth and wavefront aberrations.

## Principle of Aberrations and their Correction

**Figure 2:**
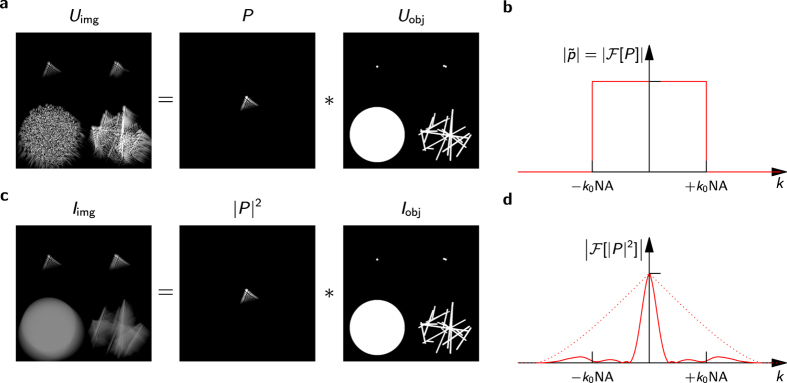
Illustration of coherent and incoherent imaging and the corresponding transfer functions. (**a**) Coherent case: the obtained image *U*_img_ is the convolution of the wave field in the object plane *U*_obj_ with the point spread function *P*; the circle (bottom-left) has laterally varying random phases. (**b**) The Fourier transform of *P*, i.e. 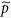, has unit magnitude within the aperture; its phase varies and results in aberrations. (**c**) Incoherent case: The intensities of the wave in the object plane *I*_obj_ convolved with the intensity of *P* give the image *I*_img_; no interference effects occur. (**d**) The incoherent transfer function is in general complex with varying magnitudes. The dotted line indicates the optimal and aberration-free incoherent transfer function.

Image formation of a single depth layer in a coherent OCT volume is described by coherent imaging theory (see e.g. ref. 23). It is assumed that the detected complex wave field in the image plane *U*_img_ is a convolution of the wave field in the object plane *U*_obj_ and the aberrated complex point spread function (PSF) *P*,


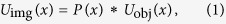


where *x* is the lateral position in the respective plane when neglecting magnification. In general, a limited aperture or aberrations will broaden or distort the PSF. The convolution with this PSF not only degrades the resolution of *U*_img_, but also causes the appearance of artificial structures by means of interference of the aberrated image points. Eventually, this introduces speckle noise if the object structures cannot be resolved (Fig. 2a).

The effect of aberrations on coherent imaging systems is even better visible in the frequency domain. If the phase of *U*_img_(*x*) is recorded correctly, the convolution theorem can be applied to the coherent imaging equation (1). It then translates to


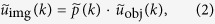


with *k* being the Fourier conjugate variable of *x*, 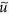 indicating the respective Fourier transforms of *U*, and 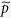 being the coherent amplitude transfer function of the imaging system, i.e., the Fourier transform of *P*. Within the aperture the coherent amplitude transfer function 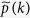 only has a phase component,


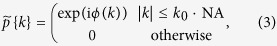


with *k*_0_ being the (center) wavenumber of the light and *ϕ*(*k*) being the phase error within the aperture. Outside the aperture, the amplitude transfer function is 0 as no light is transmitted (Fig. 2b). Hence, the multiplication with 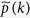 in equation (2) effectively low-pass filters the image *U*_img_. Aberrations of the imaging system including defocus will only change the phase of 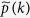, and consequently its effect on the image is completely reversed by multiplication of 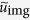 with the complex conjugate of 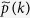, which corresponds to a deconvolution of Equation (1). Since the signal energy at all transmitted spatial frequencies is not attenuated, i.e., the absolute value of 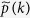 is 1 within the aperture, the reconstruction is lossless, even in the presence of noise. However, to achieve this *ϕ*(*k*) needs to be known.

The corresponding incoherent process illustrates the difference to a deconvolution in standard image processing. With an incoherent light source, only the convolution of the scattering intensities *I*_obj_ = |*U*_obj_|^2^ with the squared absolute value of the PSF is detected in the image *I*_img_,


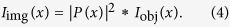


During incoherent imaging, defocus and aberrations only cause a loss in contrast for small structures (Fig. 2c), no additional interference and no speckle noise occur. However, the optical transfer function, i.e., the Fourier transform of |*P*(*x*)|^2^, is in general complex and may contain small or even zero values (Fig. 2d). Hence the effect of aberrations on image quality cannot be inverted without losing information or increasing noise. In this context, it is remarkable that a simple multiplication with the complex conjugate of 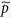 inverts the coherent process, despite of speckle noise.

The theory of coherent imaging also applies to the signal formation in FD-OCT. Here, 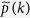 is a function of the spectral wavenumber *k*, and the Fourier conjugate to *k* is the depth. Shape and width of 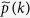 are given by the spectrum of the light source, which also determines the axial PSF and thus the resolution. Similar to coherent aberrations, an additional phase term is introduced if reference and sample arm have a group velocity dispersion mismatch or, which is relevant for FF-SS-OCT, if the sample moves axially^24^. As for aberrations, these effects are corrected losslessly by multiplication of the spectra with the conjugated phase term, if it is known.

## Aberration Detection

To computationally correct aberrations in coherent imaging, it is crucial to first determine the aberration-related phase function *ϕ*(*k*), and various approaches have been developed to determine it. One approach uses single points in the image data as guide stars^11^,^25^, which is the numerical equivalent to a direct aberration measurement with a wavefront sensor. Although photoreceptors can be used as guide stars in not too severely aberrated retinal imaging^16^, a guide star is usually not available in other retinal layers or other tissue. A second approach cross-correlates low-resolution reconstructions of the aberrated image from different sub-apertures to estimate the phase front. It worked fairly well in digital holography^26^, in FF-SS-OCT^27^, and as a rough first estimation for *in vivo* photoreceptor imaging^16^, and also to correct dispersion and axial motion in FF-SS-OCT^24^. However, these low-resolution images of scattering structures usually show independent speckle patterns, which carry no information about the aberrations and limit the precision of the phase front determination. Additionally, the uncertainty relation couples spatial resolution and accuracy of the resulting *ϕ*(*k*); increasing resolution decreases accuracy of the phase *ϕ*(*k*), and vice versa.

Here, we iteratively optimized the image quality to obtain the correcting phase, which provided very good results. Although it is computationally expensive, this idea was already applied to digital holography^28^, synthetic aperture radar (SAR, refs 21 and 29), and also scanning OCT to correct aberrations^10^ and dispersion mismatch^30^. In this approach, a wavefront *ϕ*(*k*) is assumed, and equation (2) is inverted by multiplying 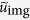 with 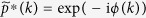. After an inverse Fourier transform a corrected image is obtained, which can be evaluated for image quality. The task is to find the *ϕ*(*k*) that gives the best quality and thus corrects aberrations.

For this approach, a metric *S*[*U*_img_(*x*)] describing image-sharpness, a parameterization of the phase error *ϕ*(*k*), and finally, an optimization algorithm are required, and their choice influences quality of the results and performance of the approach. The metric *S* needs to be minimal (or maximal) for the aberration-free and focused image, even in the presence of speckle noise. A parametrization of the phase front *ϕ*(*k*) keeps the dimensionality of the problem low and thus prevents over-fitting; still, it needs to describe all relevant aberrations. Finally, a robust optimization technique must find the global minimum of the metric without getting stuck in local minima. As the number of free parameters increases with higher aberration order, the global optimization becomes more difficult and increasingly time consuming; the algorithm performance is therefore crucial.

A variety of metrics and image-sharpness criteria have been used in previous research for coherent and incoherent imaging^8^,^21^,^29^,^31^,^32^. For a normalized complex image given by *U*_*mn*_ at pixel *m*,*n*, a special class of metrics^8^ only depends on the sum of transformed image intensities (see also Methods):


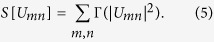


Here, we used the Shannon entropy (ref. 29) given by Γ(*I*) =−*I*log *I*, although we observed similar performance when choosing (ref. 31) Γ(*I*) = *I*^γ^ with a *γ* < 1. When these metrics reach a minimum we observed good image quality, despite of speckle dominated data.

As parametrization, the phase function *ϕ*(*k*) was expressed in Zernike polynomials. These are established in the description of optical aberrations including defocus, and their use gave good performance and results during optimization. Zernike polynomials up to 8^th^ radial degree were used, excluding piston, tip, and tilt, which results in 42 degrees of freedom.

The optimization has to find the Zernike coefficients describing *ϕ*(*k*) that give the absolute minimum of the metric *S* for the acquired data. To achieve this, we used a two-step approach. At first, a simplex-downhill algorithm was applied^33^. This algorithm follows the global trend of the metric function and is thereby rather insensitive to local minima, albeit there is no guarantee it does not converge to a local minimum. Once being close to the presumed global minimum, a gradient-based algorithm was used, which significantly boosted performance. Here, we used the conjugate gradient method^34^. A useful property of metrics described by equation (5) is that their complete gradient with respect to the Zernike coefficients can be computed efficiently; it requires only a single additional Fourier transform (see refs 8 and 21 and Methods).

If aberrations were too strong and the degrees of freedom too large, we observed that resulting images did not always show the expected structures, i.e., the optimum phase front could not be determined. We therefore modified the approach and performed the optimization first with a computationally reduced numerical aperture (see Methods). Afterwards, the optimization was repeated several times while increasing the NA. Using a smaller NA in the beginning gave more robust results since at low NA the aberrations are smaller and relatively sharp images are already obtained. Therefore, the optimization procedure detects structures it can use to converge. After increasing the NA, the optimization starts with already visible structures and converges more easily. In this way even high-order aberrations, up to 42 degrees of freedom, could be corrected for. Performance of the algorithm was judged by visually analyzing the corrected images for well known anatomical structures, such as photoreceptors, blood vessels, and nerve fiber bundles.

In general the assumption of a laterally invariant PSF is not valid, and equation (1) only holds for small volumes. The entire data were therefore divided into sufficiently small sub-regions (see Methods), which were then corrected independently. By stitching these, aberration-free data for the complete recorded volume were obtained.

## Results

**Figure 3:**
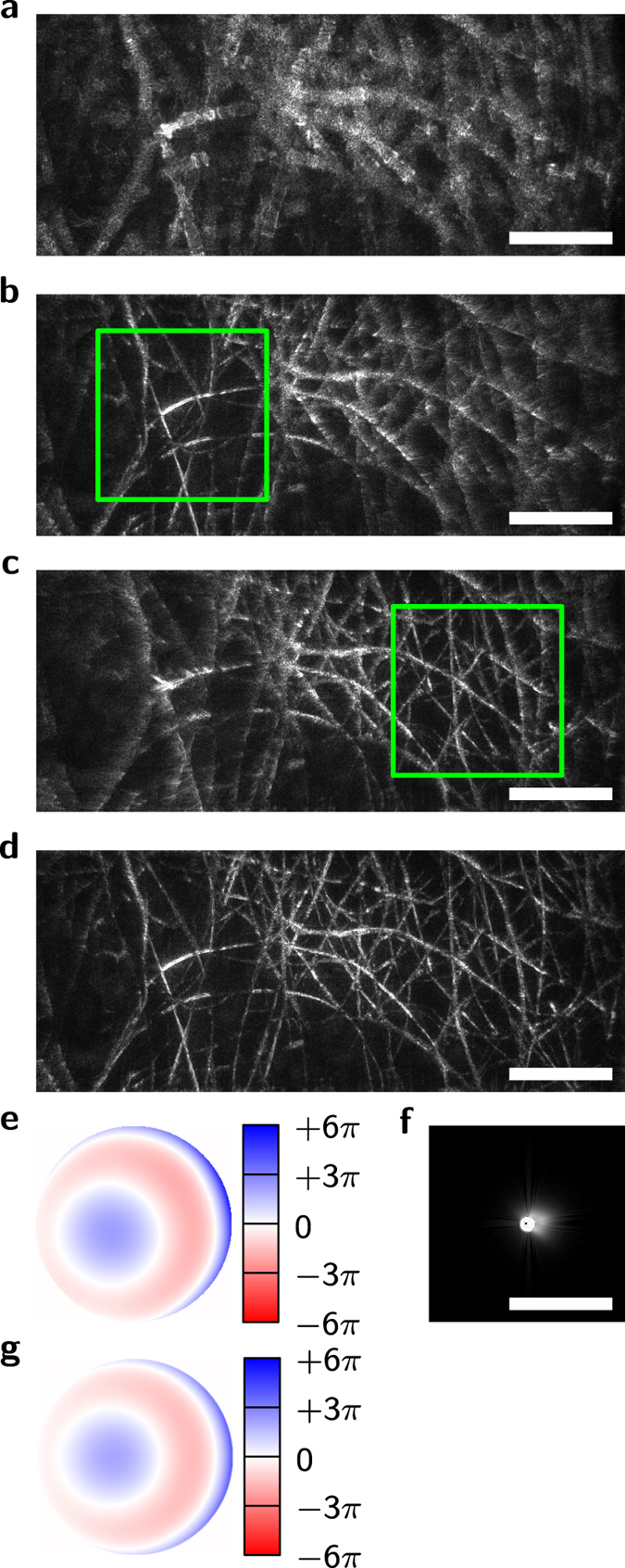
*En face* full-field swept-source OCT images from a recorded volume of lens tissue before and after aberration removal. (**a**) Before aberration correction. (**b**,**c**) After correcting aberrations for different regions (green squares). (**d**) After stitching multiple corrected regions. (**e**) Wavefront as determined for (**c**) without defocus. (**f**) Corresponding PSF. (**g**) Simulation of the expected wavefront using numerical raytracing through the objective lens. Scale bars are 0.5 mm.

To demonstrate the accuracy of the algorithms, we first imaged lens tissue (MC-5, Thorlabs) with a simple achromatic lens at an NA of 0.15, which introduced significant spherical aberrations (Fig. 3). Before correction *en face* images were severely blurred (Fig. 3a), but the optimization restored the fiber structures of the lens tissue in a certain field of view. Since the aberrations were not translation invariant, different sub-images were corrected independently (Fig. 3b,c), and by stitching these the entire image field was obtained (Fig. 3d).

Wavefront and PSF resulting from the aberration determination of Fig. 3c are shown in Fig. 3e,f, respectively. The obtained wavefront was compared to a raytracing simulation (Zemax, Fig. 3g) with almost identical results. A slight lateral misalignment of the imaging optics explains remaining differences.

**Figure 4:**
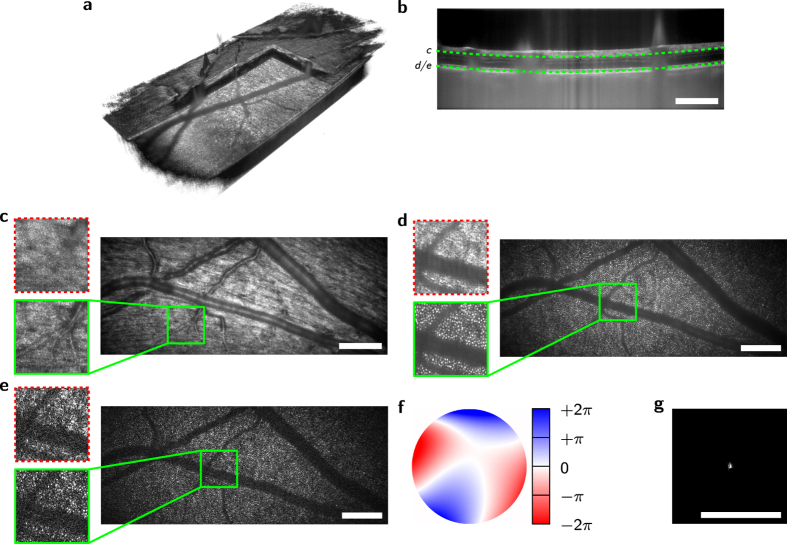
Retina volume acquired by FF-SS-OCT at NA 0.1 after aberration removal. (**a**) Rendered volume. (**b**) B-scan (sectional view) from the volume; the dashed green lines indicate the location of the curved *en face* images shown in (**c**–**e**). (**c**–**e**) Averaged (**c**,**d**) and unaveraged (**e**) aberration-corrected *en face* images and magnification of a small area in the green boxes; the same area is shown before aberration removal (dashed red boxes); nerve fiber layer (**c**) and photoreceptor mosaic (**d**) are clearly visible. (**f**) Wavefront used for aberration correction of the green box in (**d**,**e**) with defocus removed. (**g**) Corresponding PSF. Scale bars are 0.5 mm.

**Figure 5:**
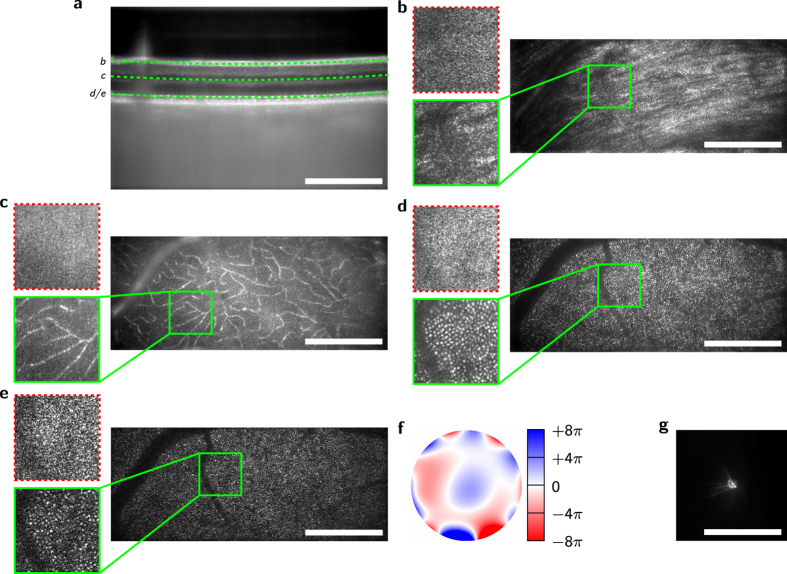
Retinal volume acquired by FF-SS-OCT at NA 0.2 corresponding to 7 mm pupil diameter after aberration removal. (**a**) B-scan (sectional view) from the recorded volume; the dashed green lines indicate the location of the curved *en face* images shown in (**b**–**e**). (**b**–**e**) Averaged (**b**–**d**) and unaveraged (**e**), aberration-corrected *en face* images with magnification of a small area in the green boxes; the same area is shown before aberration removal (dashed red boxes); nerve fiber layer (**b**), small capillaries (**c**) and photoreceptor mosaic (**d**,**e**) are only visible after correction. (**f**) Wavefront used for aberration correction of the green box in (**d**) with defocus removed. (**g**) Corresponding PSF. Scale bars are 0.5 mm.

We then imaged the retina of a young healthy volunteer *in vivo*. Two datasets were acquired, first at 14° periphery and NA 0.1 (Fig. 4), and later at 8° and 0.2 NA corresponding to 7 mm diameter of the maximally dilated pupil (Fig. 5). Without the aberration correction the volumes were laterally blurred in all layers of the retina with image degradation being significantly stronger in the high NA data, where hardly any lateral structures were visible at first. Several of the layers were aberration corrected, including the nerve fiber layer (NFL, Figs 4c and 5b), small capillaries (Fig. 5c), and the photoreceptor layer (Figs 4d,e and 5d,e). The optimization algorithm improved image quality nearly to diffraction limit, showing otherwise invisible structures. In particular, the structure of the nerve fiber layer and small capillaries became visible and single photoreceptor cells were identified.

However, the sectional images (B-scans) in Figs 4b and 5a also show a disadvantage of the FF-SS-OCT technology. In the choroid of the imaged retina, barely any structures are visible, which is caused by multiple scattered photons^35^,^36^. In addition, artifacts in the larger vessels, caused by the Doppler effect, decrease overall image quality.

The applied method for determination of the phase function *ϕ*(*k*) was robust using scattering structures and gave good results correcting the aberrations induced by the eye. Thereby, the artifacts induced by the Doppler effect, and multiple scattering did not disturb the optimization. For the measurements we performed, we observed that if structures were expected to be visible in an aberration-free imaging, the optimization was able to restore them.

The computation time with the current implementation depends on the size of the region for which aberrations are corrected. Table 1 shows processing times on a dual Intel Xeon E5620, broken down to all processing steps (see Methods) for the high NA dataset using an area of 128 × 128 pixels and 8 depth layers for correcting the aberrations. The most interesting step is the aberration determination for a small area of a single volume, which took 40 s in this case and in general ranges from a few seconds to almost a minute, depending on the chosen dataset size, the number of layers, and the number of Zernike coefficients to optimize. A significant further speed-up is expected by tweaking the reconstruction parameters, by optimizing the code, or by implementing the algorithms on a graphics processing unit (GPU).

## Discussion and Conclusion

For the first time, completely phase-stable volumetric data of human retina were acquired *in vivo*. In contrast to scanning OCT, all lateral points are acquired simultaneously, thereby preventing sample motion from causing phase changes between A-scans. Additionally, only one single laser sweep is required to obtain a volume, which removes effects from irreproducible laser sweeps. Lateral phase stability is therefore inherent to the areal data acquisition. Axial phase stability was restored by computational phase correction, resulting in axial resolution at the theoretical limit. The effective phases within a single volume were therefore only given by the tissue morphology, and phase changes between volumes consequently reflect changes thereof. Overall, the laterally phase stable detection of fields of 896 × 368 pixels was possible by a currently unmatched A-scan rate reaching 38.6 MHz, corresponding to 117 volumes per second with 84 million voxels per volume.

The phase-stable data allowed us to correct aberrations and to remove the effects of limited focal depth. The demonstrated image quality optimization was able to correct images of the nerve fiber layer, small capillaries, and photoreceptor cells. It is largely independent of the imaged structures. The chosen optimization strategy turned out to be quite robust without being overly sensitive to local minima. Having said this, there is currently no obvious way to check that the optimization found the global optimum, or that the global optimum actually corresponds to the aberration-free image. Still, the optimization was able to recover otherwise invisible or blurred structures. Only specular reflections not filling the aperture, or the absence of signals disturbed the correction. This way, in only one measurement, images of all retinal layers were obtained with near diffraction limited resolution.

Currently, the pixel number of the camera that can be used at the required acquisition rate, and the tuning range of the laser limit the system capabilities. The former restricts the field of view, the latter results in a low axial resolution of approximately 10 µm in tissue, especially when compared to the diffraction-limited lateral resolution of 2.6 µm at 0.2 NA. However, we expect that both limitations could be overcome by future technological advances.

Compared to scanning OCT and scanning laser ophthalmoscopes (SLO), a significant disadvantage of FF-SS-OCT is the sensitivity to multiple scattered photons. However, the effect was not as severe as we anticipated and high-quality images of the neuronal retina could be acquired. Furthermore, the flexibility of the system with respect to the field of view and lateral resolution is reduced, as the sampled area is bound to the camera chip, and numerical aperture and lateral sampling distance cannot be selected independently. Sensitivity of our system is lower compared to currently used scanning systems due to the higher acquisition speed. Fortunately, this lower sensitivity is partially compensated by the areal illumination, which circumvents the limitations imposed by the maximum permissible exposure of a focused beam. Additionally, incoherent background light from reflections and undesired scattering within the eye increase shot noise and reduce the achieved signal-to-noise ratio, since this light is not filtered by a confocal gating in full-field imaging. Finally, scanning OCT systems do not suffer from significant Doppler artefacts, which were visible with the proposed full-field approach. A general assessment on the advantages and disadvantages of FF-SS-OCT is difficult, since the performance depends on the imaging parameters and on the application.

Computational cost of the presented approach is not negligible. Time to completely reconstruct an entire dataset (50 volumes) and to obtain a single aberration-free layer of these volumes was more than an hour. However, starting from there, other layers can be reconstructed more easily by removing the remaining defocus.

In return for the increased computational complexity, system complexity of the FF-SS-OCT is reduced significantly compared to scanning adaptive optics (AO) OCT. No moving parts are involved. Our setup benefits from a recently available high-speed CMOS camera, which is its most advanced and expensive component and currently costs about 100,000 USD. As the technology matures, availability of suitable cameras might increase at decreasing costs. This may make FF-SS-OCT a commercially attractive alternative to complex OCT systems that implement adaptive optics (AO-OCT, e.g. refs 5 and 20) by using deformable mirrors, wavefront sensors, and closed-loop software. This mentioned complexity is still a significant hurdle for AO-OCT to find wide spread use in clinical diagnostics and research.

Fully coherent high-speed tomography does not only visualize dynamic processes with near diffraction limited resolution, but will also provide new contrast mechanisms that rely on fast but very small changes of scattering properties or optical pathlengths. Hence FF-SS-OCT can contribute to numerous medical relevant topics, e.g., measure tissue responses to photocoagulation^37^, detect heart-beat-induced pressure waves in order to probe vascular status^38^,^39^, or obtain data for optophysiology^40^.

## Methods

### Setup and data acquisition

A simple Mach-Zehnder interferometer setup was used for full-field OCT imaging of the retina (Fig. 1). Light from a tunable light source was split by a fiber coupler into reference and sample arm. The light in the reference arm was collimated and brought onto the camera via a beam splitter cube. Light from the sample arm illuminated the retina through the same beam splitter cube and the imaging optics with a parallel beam. The backscattered light from the retina was imaged onto the camera with numerical apertures (NA) ranging from 0.1 to 0.2, the latter corresponding to the maximum pupil diameter of the human eye (~7 mm), which was only achieved during mydriasis. The illuminated area on the retina was imaged onto the area-of-interest of the camera, maximizing light efficiency. Polarizations of both arms were matched to enhance interference contrast and sensitivity.

The light source (Superlum BroadSweeper BS-840-1) was tunable over 50 nm with a central wavelength of 841 nm, which resulted in about 10 µm axial resolution in tissue. In combination with a semiconductor amplifier approximately 20 mW were coupled into the interferometer, illuminating the extended area on the retina with approximately 5 mW. The laser sweep was sufficiently reproducible due to an electronically driven optoacoustic filter, and no *k*-clock was needed. The high speed CMOS camera (Photron FASTCAM SA-Z) achieved a frame rate of 60,000 fps at a resolution of 896 × 368 pixels. For each volume 512 images were acquired, each at a different wavenumber in the tuning range of the light source. The acquisition speed is thus equivalent to 38.6 MHz A-scan rate. For each measurement 50 volumes were imaged.

For optimum imaging of an *in vivo* retina, a fixation target illuminated by a green LED was used to adjust the field-of-view on the retina. The necessary steady and repeatable head position was assured by a custom fit plastic face mask, originally used in radiotherapy.

All investigations were done with healthy volunteers; written informed consent was obtained from all subjects. Compliance with the maximum permissible exposure (MPE) of the retina and all relevant safety rules was confirmed by the responsible safety officer. All experiments were performed in accordance with relevant guidelines and regulations. According to the ANSI standards, allowed radiant flux scales linearly with the size of the irradiated area^41^. Even for the smallest illuminated retinal area of about 2 mm × 0.8 mm the MPE is more than 40× higher compared to focused exposure. Though using 5 mW, a radiant flux considerably higher compared to clinical scanning OCT devices (approximately 740 μW), the exposure is still well below the MPE. The study was approved by the ethical board of the University of Lübeck (ethical approval “Ethik-Kommission Lübeck 16–080”).

### Reconstruction

To start off, a coherent average of the 50 acquired volumes was computed and the result was subtracted from all volumes. This removed fixed and phase stable artifacts in the images, while leaving the signals of the moving retina intact, since these changed from volume to volume due to eye motion. Following this, similar to FD-OCT signal processing, the OCT volumes were reconstructed by Fourier transforming the acquired 512 images along the wavenumber axis giving the depth information at each pixel of the image (*Reconstruction* in Table 1).

The data was then corrected for group velocity dispersion (GVD) mismatch in reference and sample arm and slight axial bulk motion during the wavelength sweep. Since such axial phase errors are both, of low polynomial order and equal in all A-scans of a volume, they can effectively be estimated and corrected by using the same optimization approach that facilitates the aberration correction. For this, the phase function was approximated by a polynomial of order 16 (*Correct dispersion mismatch & axial motion* in Table 1). The resulting volumes were shifted axially and laterally to maximize the correlation of the absolute values of the OCT data by using the Fourier shift theorem (*1^st^ Correlation/Alignment* in Table 1). This ensured that the layers of the retina were at identical depth positions and made selection of the input regions for the aberration correction easier. As structures were localized within each volume, the volumes could be cropped now to remove empty layers. Finally, after aberration correction, this registration process was repeated giving more precise results due to the smaller lateral structures (*2^nd^ Correlation/Alignment* in Table 1). This ensured that structures were in the same place so that absolute values of the data could be averaged over all 50 volumes. Finally, 2 to 6 depth layers of the respective retinal structures were averaged giving the presented *en face* images.

### Aberration Correction

Complex *en face* images, i.e., slices at a certain depth, intially given by *U*_0,*mn*_ at pixel *m*, *n*, were taken as input for the aberration removal step. In general multiple layers were used to improve the overall signal of the metric. Small sub-volumes of about 0.4 mm × 0.4 mm (96 × 96 pixels) with 6 to 10 layers turned out to be sufficient to correct aberrations for the low NA retinal image. For the high NA retinal images with smaller field-of-view the sub-volume size was about 0.3 mm × 0.3 mm (128 × 128 pixels) with 4 to 10 layers. In general the robustness of the algorithm increases with the sizes of the sub-volumes: The more layers and more data points are in these volumes, the fewer local minima are in the metric, at least if any structures are visible in the respective area. On the other hand, if no structures are visible in the respective layers, the data only contribute noise and should be omitted. Additionally, smaller sub-volumes are favored, since aberrations and defocus might not be constant in large volumes. Finally, computation time increases with the number of data points. Consequently, the number of chosen layers, and the number of data points in the sub-volumes, was a compromise between computational speed, robustness of the optimization, and image quality. At least in our experience the algorithm was not overly sensitive to the size of the sub-volumes that were chosen.

At first all volumes were corrected individually choosing a central region to determine the aberrations up to 8^th^ radial degree (42 degrees of freedom, *determine and correct aberrations for each volume* in Table 1). Afterwards, the volume was separated into sub-volumes, each sub-volume was corrected individually, and stitched back together; these sub-volumes overlapped with at least 16 pixels in both directions. For this last step, only aberrations to 5^th^ radial degree were taken into account and the metric was averaged over all 50 volumes (*Determine and correct aberrations for sub-regions* in Table 1). The degrees of freedom chosen in these reconstruction steps were driven by a compromise of resulting image quality and computation time. Finally, choosing more than 8^th^ radial degree may also decrease performance since difficulties in finding the global optimum can occur and more data points in the respective sub-volume may be required to increase statistics of the metric.

The aberration correction itself is similar to a process that was previously shown for synthetic aperture radar (SAR) imaging by Fienup^8^,^21^. For a given phase front *ϕ*_*μν*_, where *μ*, *ν* describe pixels in lateral frequency space, the phase front was evaluated by


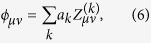


with 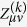 being the Zernike polynomials, *a*_*k*_ being coefficients describing the phase front, and *k* enumerating the different Zernike polynomials. The image was then reconstructed by


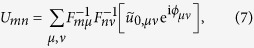


where 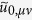 denotes the two-dimensional discrete Fourier transform of the initial, uncorrected *U*_0,*mn*_, *U*_*mn*_ the corrected image, and *F*_*mμ*_ = exp(i*mμ*/(2*πN*)) the discrete Fourier matrix. The sums can be performed by fast Fourier transformation (FFT).

The image quality or sharpness of the *en face* images (metric) was evaluated from the normalized image intensity *I*_*mn*_ = |*U*_*mn*_/∑_*m*,*n*_|*U*_*mn*_||^2^ by the Shannon entropy *S* given by


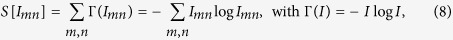


which is supposed to be minimized for optimal imaging quality^29^. In case more than one layer was used for the optimization, the metric was computed as a direct sum of the single layer metrics. Differences in focus of the respective layers were ignored and a common defocus assumed. This choice had no negative influence on the results and the algorithm recovers the mean defocus of all layers. Finally, in analogy to the work of Fienup^21^, the gradient of the metric with respect to the Zernike coefficients could be efficiently evaluated by


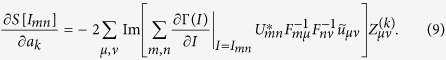


To make aberration determination more robust, we first performed the optimization for all 42 degrees of freedom at a computationally reduced NA. The aperture was reduced by masking higher spatial frequencies after Fourier transforming the complex *en face* images, which corresponds to low-pass filtering of the *en face* images. The phase correction was applied to these artificial low NA images, i.e., to the entire non-masked regions of the Fourier transform, and the metric was computed for the corresponding images. At the same time, the Zernike polynomials were always evaluated for the entire aperture and cut at the respective levels, i.e., only the inner part of each Zernike function was used. After successful determination of the Zernike coefficients for low NA images, the NA was increased and the optimization repeated with starting parameter resulting from the lower NA optimization, until the full NA was reached.

The reconstruction parameters did not have a strong influence on the obtained results and were heuristically chosen. In particular, we started at half of the maximum NA in each measurement and increased the NA in 6 equally spaced steps for the initial aberration correction until reaching the maximum NA. For the latter step, where only local aberrations were corrected, only 5 steps were used. When decreasing the number of steps or starting at a higher NA, robustness of the algorithm suffered; increasing the number of steps or starting at a slightly lower NA, however, only increased computation time. Finally, the starting NA had to be chosen sufficiently large, as the number of independent data points reduces with decreasing NA and statistical data may not be sufficient to obtain a meaningful metric. We found that the roughly used 48 × 48 pixels at numerically reduced NA were enough when averaging the metric over 6 layers.

### Implementation

The code for the reconstruction was implemented in C++ and compiled with the GNU compiler collection (GCC). OpenMP was used for partial parallelization. Furthermore, to compute the fast Fourier transform the FFTW library was used^42^. For the optimization strategies the GNU Scientific Library (GSL) was applied^43^. Although run-time performance was taken into account, the code was not fully optimized. Parameters for the reconstruction, such as the number of layers taken into account or the number of reduced NAs, were chosen conservatively to reach a robust reconstruction and sacrificing computing speed. Hence, performance can probably be increased by choosing parameters more carefully.

## Additional Information

**How to cite this article**: Hillmann, D. *et al.* Aberration-free volumetric high-speed imaging of *in vivo* retina. *Sci. Rep.* **6**, 35209; doi: 10.1038/srep35209 (2016).

## Figures and Tables

**Table 1:** Computation time required to reconstruct a dataset, broken down to each processing step as measured for the high NA dataset.

	Single volume	50 Volumes (Single dataset)
Reconstruction	2.5 s	140 s
Correct dispersion mismatch & axial motion	4.3 s	215 s
1^st^ Correlation/Alignment	—	682 s
Determine aberrations (each volume)	40 s	2013 s
Correct aberrations	14 s	684 s
2^nd^ Correlation/Alignment	—	357 s
Determine aberrations (1 sub-region)	—	43 s
Determine aberrations (all 40 sub-regions)	—	1735 s
Correct aberrations (all 40 sub-regions)	—	443 s

In this case 8 layers in an area of 128 × 128 pixels in each sub-region were taken into account when computing the metric. The computation time for a full dataset (50 volumes) sums up to about 1.7 h from camera raw data to processed dataset. Disk input-output was not counted, i.e., it is assumed all data resides in random access memory (RAM). For a detailed description of the processing steps, see Methods.
